# Risk Factors for SARS Transmission from Patients Requiring Intubation: A Multicentre Investigation in Toronto, Canada

**DOI:** 10.1371/journal.pone.0010717

**Published:** 2010-05-19

**Authors:** Janet Raboud, Altynay Shigayeva, Allison McGeer, Erika Bontovics, Martin Chapman, Denise Gravel, Bonnie Henry, Stephen Lapinsky, Mark Loeb, L. Clifford McDonald, Marianna Ofner, Shirley Paton, Donna Reynolds, Damon Scales, Sandy Shen, Andrew Simor, Thomas Stewart, Mary Vearncombe, Dick Zoutman, Karen Green

**Affiliations:** 1 Division of Infectious Diseases, University Health Network, Toronto, Ontario, Canada; 2 Dalla Lana School of Public Health, University of Toronto, Toronto, Ontario, Canada; 3 Mount Sinai Hospital, Toronto, Ontario, Canada; 4 Department of Laboratory Medicine and Pathology, University of Toronto, Toronto, Ontario, Canada; 5 Ontario Ministry of Health and Long Term Care, Toronto, Ontario, Canada; 6 Department of Anesthesia, University of Toronto, Toronto, Ontario, Canada; 7 Sunnybrook Health Sciences Centre, Toronto, Ontario, Canada; 8 Public Health Agency of Canada, Ottawa, Ontario, Canada; 9 British Columbia Centre for Disease Control, Vancouver, British Columbia, Canada; 10 Department of Medicine, University of Toronto, Toronto, Ontario, Canada; 11 Department of Pathology and Molecular Medicine, McMaster University, Hamilton, Ontario, Canada; 12 Centers for Disease Control and Prevention, Atlanta, Georgia, United States of America; 13 Durham Region Health Department, Whitby, Ontario, Canada; 14 Department of Microbiology and Immunology, Queen's University, Kingston, Ontario, Canada; U.S. Naval Medical Research Center Detachment/Centers for Disease Control, United States of America

## Abstract

**Background:**

In the 2003 Toronto SARS outbreak, SARS-CoV was transmitted in hospitals despite adherence to infection control procedures. Considerable controversy resulted regarding which procedures and behaviours were associated with the greatest risk of SARS-CoV transmission.

**Methods:**

A retrospective cohort study was conducted to identify risk factors for transmission of SARS-CoV during intubation from laboratory confirmed SARS patients to HCWs involved in their care. All SARS patients requiring intubation during the Toronto outbreak were identified. All HCWs who provided care to intubated SARS patients during treatment or transportation and who entered a patient room or had direct patient contact from 24 hours before to 4 hours after intubation were eligible for this study. Data was collected on patients by chart review and on HCWs by interviewer-administered questionnaire. Generalized estimating equation (GEE) logistic regression models and classification and regression trees (CART) were used to identify risk factors for SARS transmission.

**Results:**

45 laboratory-confirmed intubated SARS patients were identified. Of the 697 HCWs involved in their care, 624 (90%) participated in the study. SARS-CoV was transmitted to 26 HCWs from 7 patients; 21 HCWs were infected by 3 patients. In multivariate GEE logistic regression models, presence in the room during fiberoptic intubation (OR = 2.79, p = .004) or ECG (OR = 3.52, p = .002), unprotected eye contact with secretions (OR = 7.34, p = .001), patient APACHE II score ≥20 (OR = 17.05, p = .009) and patient Pa0_2_/Fi0_2_ ratio ≤59 (OR = 8.65, p = .001) were associated with increased risk of transmission of SARS-CoV. In CART analyses, the four covariates which explained the greatest amount of variation in SARS-CoV transmission were covariates representing individual patients.

**Conclusion:**

Close contact with the airway of severely ill patients and failure of infection control practices to prevent exposure to respiratory secretions were associated with transmission of SARS-CoV. Rates of transmission of SARS-CoV varied widely among patients.

## Introduction

On March 7, 2003, a son of Canada's index SARS case was admitted to a hospital in Toronto with a diagnosis of community-acquired pneumonia. Because he and other family members were not identified as infected with SARS CoV until March 13, infection was transmitted to patients, volunteers, visitors and health care workers in this community hospital, and subsequently in other hospitals and the community throughout Greater Toronto Area (GTA). Over the next three months, SARS-CoV would be transmitted to 375 persons in Toronto, 271 (72%) of whom acquired their infections in health care settings.[1]–[3] The healthcare workers at greatest risk of acquiring SARS were those caring for critically ill SARS patients, and transmission was documented despite the use of recommended personal protective equipment. [3]–[7]

During and after the outbreak, considerable controversy evolved regarding how HCWs using precautions became infected, and what care activities and/or behaviours posed the greatest risk of transmission. We conducted a retrospective cohort study designed to identify risk factors associated with transmission of SARS-CoV from patients requiring intubation to HCWs involved in their care. In particular, we wanted to assess the risk of SARS-CoV transmission associated with adherence to infection control precautions and with performance of “high-risk” procedures in a setting in which adjustment for potential patient-related characteristics was possible.

## Methods

### Ethics Statement

Patient clinical details were obtained through review of health records which was conducted for a study of clinical presentation and management of SARS. The study was approved by the IRB of each hospital where patients were treated. Individual consents were not obtained which is the practice for chart review studies in which individual patient identifying information is not required. All patients in the SARS outbreak were given a unique identifying number and all data were coded with this unique number. There was no information collected that could identify the patient personally.

HCWs blood samples were obtained through a seroprevalence study, which was part of the public health investigation and received IRB approval at each hospital where HCWs were enrolled. HCWs provided consent for blood samples.

Approvals for both studies were obtained from The Mount Sinai Hospital Research Ethics Board as well as the Research Ethics Boards/Committees of the following institutions: Sunnybrook Health Center. North York General Hospital, The Scarborough Hospital, Rouge Valley Health Care, Humber River Regional Hospital, Markham Stuffily Hospital, St. Michael's Hospital, St. Joseph's Health Center, Southlake Regional Health Center, Toronto East General Hospital, University Health Center, and Lakeridge Health Center.

HCWs were interviewed as part of a public health investigation into the transmission of SARS-CoV. HCWs were invited to participate and consent was implied by willingness of HCWs to be interviewed about their experiences. No IRB approval was required as these interviews occurred as part of the public health outbreak investigation which is a legislated responsibility of the Ontario public health units.

### Identification and Classification of Patients and HCWs

#### Patients

SARS patients requiring intubation were identified by reviewing outbreak line lists from the Province of Ontario, local public health units, and hospitals. All cases in the Toronto outbreak who met the clinical and epidemiologic criteria for SARS [8] and who required intubation were included. Clinical criteria for SARS included fever >38°C, one or more respiratory symptoms, radiological evidence of pneumonia or respiratory distress syndrome and no alternative diagnosis; or having an unexplained acute respiratory illness resulting in death between March 5, 2003 and June 12, 2003. [8] Epidemiological criteria included: visiting a setting that was associated with a SARS cluster or having cared for, lived with or had face-to-face contact with a person known to have SARS in the 10 days prior to onset of symptoms.

All patients in whom SARS was suspected had multiple clinical specimens tested by culture and PCR for SARS-CoV, including nasal swabs, nasopharyngeal swabs, throat swabs, sputum/endotracheal secretions, conjunctival swabs, stool, urine and, if available, bronchoalveolar lavage and post-mortem lung and other tissue samples. [9] If possible, acute and convalescent serum were also obtained. Patients were classified as laboratory confirmed, probable, unclassifiable or not a case of SARS-associated coronavirus (CoV) disease, based on laboratory testing. Laboratory confirmed cases were those with SARS-CoV antibodies detected in serum obtained after the onset of symptoms by tests conducted in two different reference laboratories, SARS CoV isolated by cell culture from a clinical and/or autopsy specimen, SARS-CoV RNA detected by reverse transcription polymerase chain reaction (RT-PCR) from an autopsy specimen with compatible histological findings, or SARS-CoV RNA detected by RT-PCR from at least two different specimens by two different reference laboratories. [9] Probable cases were those in whom a convalescent serum specimen was not available for testing and other laboratory criteria for SARS were not met, but who had clinically compatible disease and at least one household contact who was a laboratory confirmed case of SARS. Cases were unclassifiable if they did not meet criteria for laboratory confirmed disease, and either had no household contacts, or contacts without laboratory confirmed disease. Patients were classified as not having SARS if antibodies to SARS-CoV could not be detected in serum obtained more than 28 days after symptom onset.

#### HCWs

All HCWs who provided care to intubated SARS patients during treatment or transportation, or who entered the room of such patients from 24 hours prior to intubation until 4 hours after intubation were eligible for this study, and approached for consent to participate. They were identified by review of patient charts, work schedules, assignments and on-call schedules, and by asking individuals being interviewed to recall which other staff members were present.

We reviewed outbreak line lists and interviewed HCWs to identify those with fever or respiratory symptoms with onset from 1 to 14 days after each work shift with identified patients. Participating HCWs were also asked to submit a convalescent serum sample for SARS-CoV antibody testing. HCWs were classified as SARS if they had antibodies to SARS-CoV detected in their convalescent serum, or if they met the SARS outbreak case definition [8] and did not have serology performed. They were classified as not having SARS if they had negative serology or if they did not have serology done and had no fever or respiratory symptoms. They were deemed unclassifiable if they had SARS compatible symptoms that did not meet the case definition and convalescent serology was not available.

Staff were asked about their history of travel to areas affected by SARS, care provided to any possible SARS patients, and household and other contact with potential SARS cases, including other HCWs. For symptomatic HCWs, we recorded potential exposures in the period from 24 hours to 12 days before onset of illness using outbreak contact tracing data, interviews and work assignments. For HCWs who were SARS-CoV seropositive without symptoms, we reviewed all potential exposures which occurred during the outbreak period.

A high risk exposure to SARS was defined as being in the same room as or involved in the transport of a SARS patient either during the 24 hours prior to the patient requiring intubation, or at any time when adequate precautions had not been implemented. Adequate precautions were defined as the patient being in a negative pressure room and gown, gloves, mask (surgical mask or, N95 or higher respirator) and eye protection (goggles, safety glasses or face shield) being worn.

#### Cohorts for analysis

The primary analysis was restricted to patients with laboratory-confirmed SARS, and HCWs who were either asymptomatic or had laboratory confirmed disease and whose only high risk exposure was contact with intubated patients. A secondary analysis included probable and laboratory confirmed SARS patients and all infected HCWs. Because there were no significant differences between the two analyses, only the results of the primary analysis are presented here.

### Measurements

#### Patients

A standardized data collection form was used for chart review, and included: age, sex, underlying chronic conditions, date of symptom onset, duration of illness at the time of intubation, date of hospital and ICU admission, date of discharge, outcome; documented vomiting, diarrhea, incontinence, agitation, and combativeness during the 24 hours prior to intubation, acute physiology and chronic health evaluation II score during the same period (APACHE II) [10], lowest PaO_2_ to FiO_2_ (P/F ratio) ratio during the first 24 hours in the ICU and maximum inspired oxygen requirement (FiO_2_) on the second day of hospital admission. We also recorded whether attending physicians suspected that the patient had SARS at the time of intubation.

Details of the intubation procedures were obtained from medical records and interviews with the staff member who performed the intubation. These included: method of intubation (fiber optic, laryngoscopic, nasopharyngeal, or tracheotomy), patient combativeness, number of attempts required, experience of person performing the procedure, and whether manual intubation was required before and/or after intubation. Intubations were classified as difficult if more than one attempt was required, if fiber optic visualization or any adjuvant device for difficult airway was required, or if the patient was combative. HCWs performing intubations were considered experienced if they had more than three years of experience and performed more than one intubation per month, or if they had more than one year of experience and performed more than one intubation per week.

Procedures and activities performed during the defined exposure period were documented. These included: airway management procedures (oxygen therapy, bronchoscopy, non-invasive positive pressure ventilation, manual ventilation, suctioning, type of mechanical ventilation, nebulizer treatment) and cardiopulmonary resuscitation and defibrillation and other care activities (chest x-ray; electrocardiogram; chest tube insertion; insertion of central venous, peripheral or arterial catheters; insertion of urinary catheter or nasogastric tube; collection of blood, urine, stool, or sputum samples).

#### HCWs

A structured questionnaire was administered to HCWs through a face-to-face interview or by telephone interview if a respondent was in quarantine or had left their job. HCWs were asked about occupation, years of experience in their current occupation, age, sex, history of smoking and underlying chronic conditions.

All non-demographic variables were assessed separately for each eligible shift. An eligible shift was defined as one in which a HCW provided care for one patient during that patient's defined exposure period. When HCWs worked two shifts during one exposure period, data from these two shifts were combined for the analysis.

For each shift, questions were asked about the number of room entries, cumulative time spent in a patient room, and type and duration of contact with a patient. We also assessed level of involvement (performed, assisted or observed) in and the amount of time the HCW spent in the patient's room during 34 patient care activities, as well as the HCW's presence in the patient's room while the patient was receiving non-invasive ventilation, oxygen therapy, and mechanical ventilation. Copies of charts were provided to interviewees to assist in recall.

Type of personal protection equipment (PPE) -gloves, gown, goggles or face shield, surgical mask, N95 or higher respirator, and frequency of its use (never, sometimes, most of the time and always) during the shift were assessed. We also asked about PPE use separately during involvement in patient care activities and categorized HCWs' sequence of removal of PPE based on the potential risk of self-contamination of mucous membranes. [11]

Participants were asked whether they had received SARS-specific infection control training prior to their eligible shift. Infection control training was categorized as active (face-to-face teaching) or passive (written instructions only). Exposure incidents that occurred during the eligible shift were recorded, including needle stick injuries and exposure of skin or mucous membranes to patient's body fluids, blood, secretions or mucous membranes.

#### Laboratory tests

Laboratory testing was conducted in collaboration with the Ontario Laboratory Working Group for the Rapid Diagnosis of Emerging Diseases, the Central Ontario Public Health Laboratory, the SARS autopsy investigation, the British Columbia Centre for Disease Control, and the Canadian National Microbiology Laboratory. Detection of IgG antibody to SARS-CoV was performed by using an enzyme-linked immunosorbent assay (ELISA) or immunofluorescent assay (IFA) as previously described. [9]

### Statistical Methods

Demographic and clinical characteristics were summarized with counts and percentages for categorical variables and with medians and interquartile ranges for ordinal and continuous variables. These characteristics were compared between patients who did and did not transmit SARS-CoV and between HCWs who did and did not develop SARS with chi square or Fisher's exact tests for categorical variables and Wilcoxon rank sum tests for ordinal and continuous variables.

Two statistical methods were used to identify factors associated with SARS-CoV transmission: Classification and regression trees (CART) [12] and Generalized Estimating Equation (GEE) models. [13] CART is a non-parametric method of identifying predictor variables by using binary recursive partitioning: subsets of patients are formed by examining each possible cut point of each variable to identify the cut point that resulted in maximum discrimination between subgroups of patients with respect to the probability of acquiring SARS. CART analyses were conducted twice: with and without allowing patient specific covariates to be predictor variables.

GEE logistic regression models were used to identify predictor variables while adjusting for correlation among responses from HCWs caring for the same patient. Correlation within HCW shifts caring for a single patient was assumed to follow the exchangeable correlation structure. Correlation among shifts worked by the same HCW was not modeled. Covariates which had a p value <0.10 in univariate GEE logistic regression models were considered as candidates for entry into the multivariable GEE model.

## Results

Fifty-six (15%) of 360 SARS patients who received treatment in one of 20 Ontario hospitals required intubation. Eleven patients were excluded from the primary analysis: seven had probable SARS, one patient was unclassifiable and three patients had at least one specimen positive by PCR, but did not have detectable antibodies to SARS CoV at ≥28 days after symptom onset. Thus, 45 patients were classified as having laboratory confirmed SARS and were included in the primary analysis as potential index cases for the exposed HCWs.

Overall, 624 (90%) of 697 HCWs who were identified as having provided care to the 45 laboratory confirmed SARS patients consented to participate, could be classified as having had SARS or not, and had no other high risk exposures. They worked a total of 786 eligible shifts. Interviews were completed at a median of 4.2 months (range 0.2–10 months) after the eligible shift. 111 (17.8%) HCWs were involved in the care of more than one intubated SARS patient, with a maximum of 8 eligible patients cared for by the same HCW. The median number of participating HCWs involved in the care of each SARS patient during the relevant time period (24 hours prior to intubation until 4 hours post-intubation) was 15 (range 6 to 30).

Of the 624 participating HCWs, 26 contracted SARS; all survived and none required intubation. SARS-CoV transmission to HCWs was attributable to 7 of the 45 laboratory confirmed SARS patients. Transmission to 22 HCWs could be definitively attributed to a single patient, with 6 patients transmitting to 1, 1, 2, 5, 6 and 7 HCWs respectively. The remaining four HCWs who acquired SARS had cared for more than one SARS patient during the high risk period, making it difficult to precisely identify which patient was the source of infection. For the primary analysis, we assumed a ‘most likely scenario’ for assigning transmission to one patient. Three of the infected staff members cared for two patients whose intubations were a few hours apart. Since one patient was the source of infection for five other individuals; we assumed in the primary analysis that this patient had also transmitted SARS-CoV to these 3 HCWs. The fourth HCW was involved in the care of two intubated patients; transmission was assumed to have occurred on the shift that involved emergency intubation and cardiac resuscitation.

No patient characteristics were statistically significantly different between patients who did and did not transmit SARS-CoV (Table 1). HCWs who contracted SARS were more likely to be paramedics (p<.01) and had less infection control training (p<.009) than other workers (Table 2). They were less likely to always wear goggles (p<0.01) or a gown (p = .02) while in the patient's room, and more likely to have used less effective methods of respiratory protection while in a patient's room (p = .04). They were more likely to have participated in administering non-invasive ventilation (p<0.01), and to have performed ECGs (p<0.01), fiber optic intubation (p<0.01) or manual ventilation before intubation (p<0.01) than HCWs who did not develop SARS (Table 3).

**Table 1:** Demographic and clinical characteristics of SARS patients requiring intubation in the Toronto outbreak by presence or absence of transmission to HCWs.

	No Transmission of SARS to HCWs N = 38	SARS Transmission to at least one HCW N = 7	p value	All N = 45
**Patient Characteristics at Admission**				
Age, years^a^	59 (45, 72)	63 (47, 75)	0.64	61 (47, 72)
Sex, number (%) male	17(45%)	5 (71%)	0.24^b^	22 (49%)
Chronic underlying illness^c^	15(39%)	3(43%)	0.99^b^	18 (40%)
Diabetes	12(32%)	3(43%)	0.67^b^	15(33%)
Immunosuppression	3(8%)	1(14%)	0.50^b^	4(9%)
PaO_2_/FiO_2_ ratio^a^	84 (67, 116)	85 (51, 112)	0.64	84 (59, 112)
FiO_2_ on day 2 of hospitalization^a^	0.38 (0, 0.95)	0.4 (0, 1)	0.78	0.4 (0, 0.95)
Apache II score (1^st^ 24 h ICU)^a^	16 (14, 20)	21 (11, 22)	0.20	16 (14,21)
Patient transmitted SARS prior^d^	15(39%)	4(57%)	0.43^b^	19 (42%)
**Patient Characteristics at Intubation**				
Day of illness at time of intubation^a^	9(7, 13)	7(7, 9)	0.20	9(7, 12)
Diarrhea 24 hours prior to intubation	18(47%)	1(14%)	0.21^b^	19 (42%)
Vomiting 24 hours prior to intubation	7(18%)	1(14%)	0.99^b^	8(18%)
Copious secretions at intubation	14(37%)	1(14%)	0.40^b^	15(33%)
Combative during intubation	7(18%)	1(14%)	0.99^b^	8(18%)
Intubated in negative pressure room	33(87%)	6(86%)	0.99^b^	39(87%)
Patient recognized as SARS at time of intubation	33 (87%)	6(86%)	0.99^b^	39 (87%)
**Characteristics of Intubation**				
Intubation difficult	12(32%)	3(43%)	0.67^b^	15(33%)
Intubation performed during night shift	7(18%)	1(14%)	0.99^b^	8(18%)
Primary intubator experienced	32(84%)	6(86%)	0.99^b^	38(84%)
Intubation emergent	0(0%)	1(14%)	0.16^b^	1(2%)
**Patient Outcome**				
Deceased	18 (47%)	5 (71%)	0.40^b^	23 (51%)

a Values are given as median (lower quartile, upper quartile).

b Fisher's exact test.

c Chronic underlying illness is defined as having one or more of diabetes, chronic renal failure, chronic liver disease, chronic obstructive pulmonary syndrome, coronary artery disease, congestive heart failure, active cancer, HIV/AIDS, transplantation.

d Transmission occurred from patient to household or hospital contact prior to study period (starting 24 hours prior to intubation).

**Table 2:** Characteristics of health care workers who provided care to intubated SARS patients in Toronto, by SARS acquisition status.

	HCWs who did not develop SARS N = 598	HCWs who developed SARS N = 26	p value	All N = 624
Age, years^a^	40 (34, 48)	38.5 (33, 44)	0.21	40 (34, 47)
Sex, number (%) male	145 (24%)	10 (38%)	0.10	155 (25%)
Had chronic disease^b^(N = 609)	36 (6%)	1 (4%)	0.99^c^	37 (6%)
PositionStaff physicianMedical resident/internRegistered nurseRespiratory therapistRadiology technologistHousekeeperPersonal service assistantLaboratory technician/technologistParamedic/emergency medical technicianPharmacistWard clerkPorterPhysiotherapist/occupational therapistOther	73 (12%)14 (2%)272 (45%)85 (14%)66 (11%)38 (6%)25 (4%)14 (2%)0 (0%)2 (0.3%)2 (0.3%)2 (0.3%)1 (0.2%)4 (0.7%)	4 (15%)2 (8%)11 (42%)4 (15%)1 (4%)0 (0%)1 (4%)0 (0%)3 (12%)0 (0%)0 (0%)0 (0%)0 (0%)0 (0%)	0.55^c^0.14^c^0.750.78^c^0.34^c^0.39^c^0.99^c^0.99^c^<0.01^c^0.99^c^0.99^c^0.99^c^0.99^c^0.99^c^	77 (12%)16 (3%)283 (45%)89 (14%)67 (11%)38 (6%)26 (4%)14 (2%)3 (0.5%)2 (0.3%)2 (0.3%)2 (0.3%)1 (0.2%)4 (0.6%)
Total hours worked in 7 days prior to study period^a^ (N = 565)	37.5(32, 48) (N = 545)	40(36, 55) (N = 20)	0.15	38(32, 48)
Always wore goggles while in patient room	451(75%)	13(50%)	<0.01	464 (74%)
Always wore gloves while in patient room	555 (93%)	23 (88%)	0.43^c^	578 (93%)
Always wore gown while in patient room	541 (90%)	20 (77%)	0.04^c^	561 (90%)
Respiratory protection while in patient room 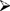 None 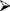 Surgical mask 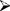 N95 or equivalent 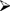 Higher protection than N95(e.g., N95 plus Stryker hood, PAPRs)	49 (8%)25 (4%)496 (83%)28 (5%)	3 (12%)5 (19%)18 (69%)0 (0%)	0.04^d^	52 (8%)30 (5%)514 (82%)28 (4%)
Personal protective equipment removal^e^ 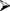 None used 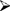 No hand hygiene performed 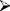 No hand hygiene before removing face protection, hand hygiene at the end 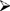 Hand hygiene before removing face protection, no hand hygiene at the end 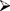 Hand hygiene before removing face protection, plus hand hygiene at the end	41 (7%)192 (32%)290 (48%)14 (2%)61 (10%)	3 (12%)11 (42%)8 (31%)0 (0%)4 (15%)	0.56^d^	44 (7%)203 (33%)298 (48%)14 (2%)65 (10%)
Infection control training 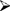 None 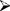 Other (information from colleagues) 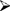 Email or written instructions 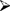 Group sessions 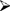 Individual face to face instruction	173 (29%)9 (2%)136 (23%)127 (21%)153 (26%)	16 (62%)0 (0%)2 (8%)2(8%)6 (23%)	0.009^d^	189 (30%)9 (1%)138 (22%)129 (21%)159 (25%)

a Values are given as median (lower quartile, upper quartile).

b Chronic underlying illness is defined as having one or more of diabetes, chronic renal failure, chronic liver disease, chronic obstructive pulmonary syndrome, coronary artery disease, congestive heart failure, active cancer, HIV/AIDS, transplantation.

c Fisher's exact test.

d Cochran-Armitage test for trend.

e see reference 11.

**Table 3:** HCW participation in patient care procedures, by SARS acquisition status.

	HCWs who did not develop SARS N = 598	HCWs who developed SARS N = 26	p value	All N = 624
**Potential HCW exposure to respiratory secretions** a				
Non-invasive ventilation	99 (17%)	10 (38%)	<0.01	109 (17%)
High flow oxygen	106 (18%)	2 (8%)	0.29^b^	108 (17%)
Mechanical ventilation	227 (38%)	9 (35%)	.73	236 (38%)
**HCW involvement in intubation** a				
Intubation (including fiber optic intubation)	132 (22%)	12 (46%)	<0.01	144 (23%)
Suctioning before intubation	106 (18%)	7 (27%)	0.29^b^	113 (18%)
Suctioning after intubation	155 (26%)	10 (38%)	0.16	165 (26%)
Manual ventilation before intubation	108 (18%)	10 (38%)	0.02^b^	118 (19%)
Manual ventilation after intubation	114 (19%)	6 (23%)	0.61	120 (19%)
**Procedures with potential exposure to respiratory secretions** a				
Cardiac compressions	8 (1%)	1 (4%)	0.32^b^	9 (1%)
Bronchoscopy	10 (2%)	0 (0%)	0.99^b^	10 (2%)
Chest physiotherapy	47 (8%)	1 (4%)	0.71^b^	48 (8%)
Defibrillation	3 (1%)	1 (4%)	0.15^b^	4 (1%)
Collection of sputum sample	38 (6%)	4 (15%)	0.09^b^	42 (7%)
Nebulizer treatment	9 (2%)	0 (0%)	0.99^b^	9 (1%)
Manipulation of oxygen mask	280 (47%)	17 (65%)	0.06	297 (48%)
Insertion of NG tube	45 (8%)	2 (8%)	0.99^b^	47 (8%)
**Procedures with potential exposure to stool or urine** a				
Collection of stool sample	17 (3%)	2 (8%)	0.19^b^	19 (3%)
Emptying urine bag or taking urine sample	137 (23%)	4 (15%)	0.37	141 (23%)
Emptying bed pan	48 (8%)	1 (4%)	0.71^b^	49 (8%)
**Other procedures** a				
Insertion of central venous line	53 (9%)	3 (12%)	0.72^b^	56 (9%)
Insertion of urinary catheter	38 (6%)	3 (12%)	0.24^b^	41 (7%)
Insertion of peripheral IV access line	138 (23%)	7 (27%)	0.65	145 (23%)
Venipuncture/arterial blood gas	160 (27%)	7 (27%)	0.99	167 (27%)
Chest tube insertion	12 (2%)	0 (0%)	0.99^b^	12 (2%)
ECG	98 (16%)	11 (42%)	<0.01^b^	109 (17%)
Bathing a patient	133 (22%)	4 (15%)	0.41	137 (22%)
Feeding a patient	87 (15%)	1 (4%)	0.16^b^	88 (14%)
Transporting a patient	93 (16%)	7 (27%)	0.17^b^	100 (16%)
Taking oral temperature	71 (12%)	2 (8%)	0.76^b^	73 (12%)
Administering oral medication	111 (19%)	3 (12%)	0.45^b^	114 (18%)
**Housekeeping activities** a				
Cleaning equipment	150(25%)	7(27%)	0.83	157 (25%)
Cleaning room	81(14%)	2(8%)	0.56^b^	83 (13%)
Cleaning bathroom	41(7%)	1(4%)	0.99^b^	42 (7%)
Changing bedding	171(29%)	7(27%)	0.85	178 (29%)

a For these potential risk factors, health care workers were considered exposed if they reported being in the room while the patient was receiving the therapy.

b Fisher's exact test.

**Figure 1:**
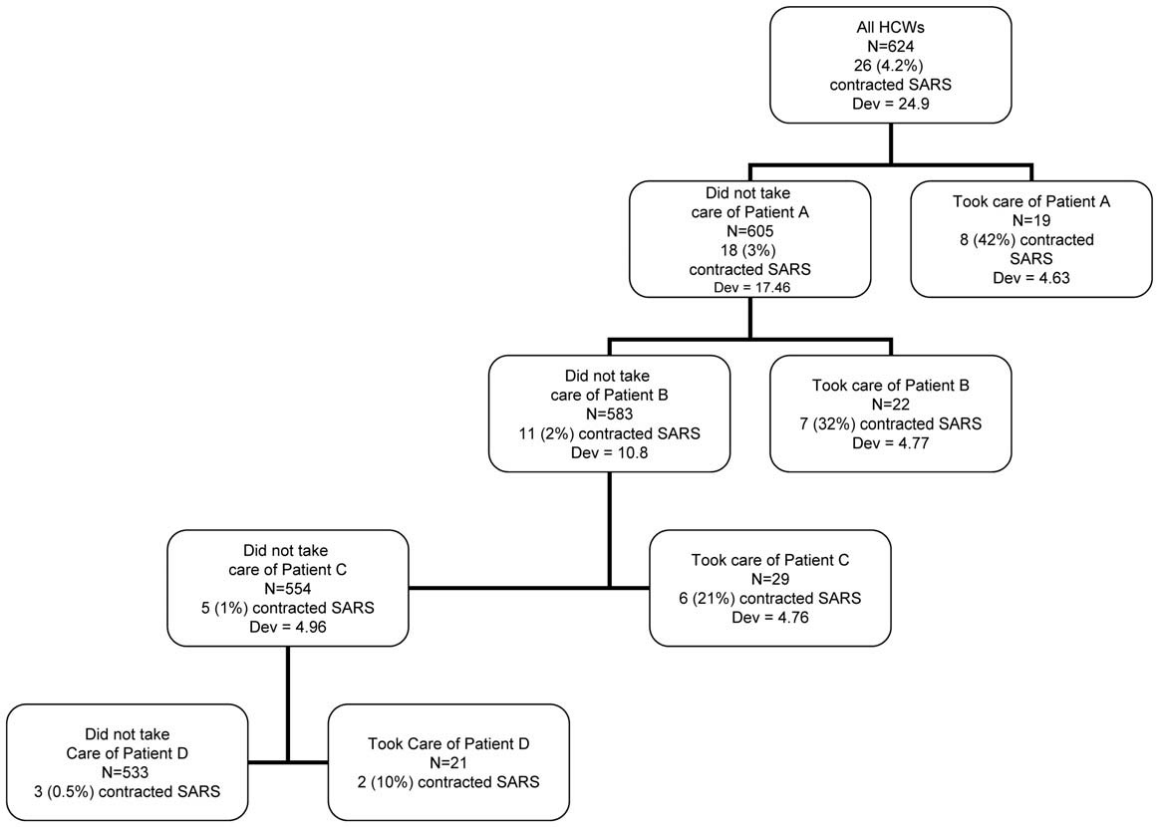
Classification and regression tree analysis of risk factors for SARS transmission, allowing patient-specific covariates.

**Figure 2:**
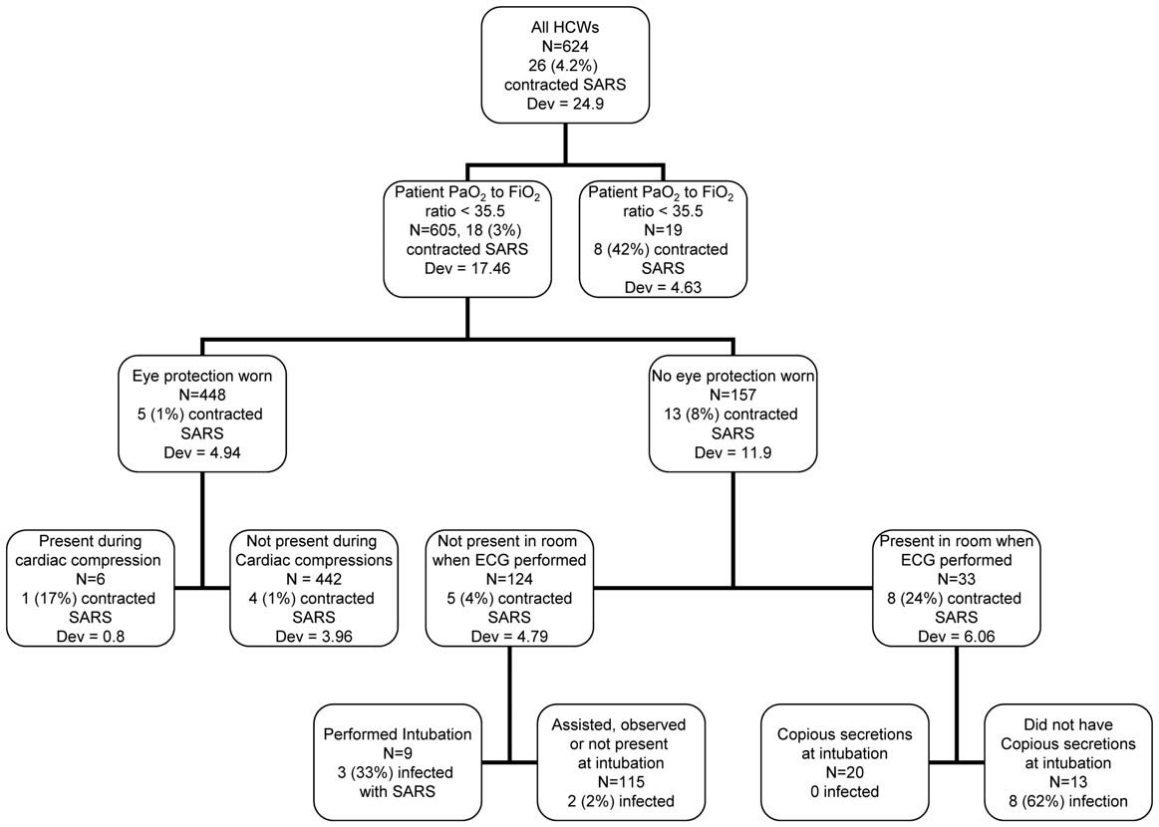
Classification and regression tree analysis of risk factors for SARS transmission, not allowing patient specific covariates.

In the CART analysis allowing individual patients to be entered as covariates, patient covariates were the top four splitting variables according to the deviance at each node (Figure 1). Twenty-three of the 26 HCWs who acquired SARS were involved in the care of one of these four patients. The results of this analysis did not change when only HCWs with exposures to single patients were considered, or when we altered the assumptions regarding which patient infected the four HCWs with exposures to two patients. If patients were not included as predictor variables (Figure 2), then the first splitting variable was whether the patient P/F ratio was >35.5 or <35.5 (3% vs 42% contracted SARS). Among the 605 HCWs involved in the care of patients with P/F ratio >35.5, the most predictive variable was whether or not the HCW wore eye protection (1% of HCWs infected wearing eye protection vs 8% infected not using eye protection). It should be noted that the single patient with P/F ratio <35.5 was the patient who was the first splitting variable in the CART analysis with individual patients included.

In univariate GEE models (Table S1), patient characteristics associated with transmitting SARS-CoV were: P/F ratio ≤59, gender, death due to SARS, APACHE II score ≥20, presence of diarrhea within 24 hours of intubation, any chronic underlying illness disease, and diabetes mellitus (when underlying illnesses were considered separately). HCW characteristics associated with acquiring SARS included eye/mucous membrane exposure to blood/body fluids, performing intubation, and presence in the room for any of cardiac compressions, defibrillation, ECG, intubation, manual ventilation, manipulation of oxygen mask or tubing, collection of sputum or stool samples, or transporting the patient. In the multivariate GEE logistic regression model (Table 4), the independent predictors were eye or mucous membrane exposure to body fluids (OR = 7.34, p = .001), patient APACHE II score ≥20 (OR = 17.05, p = .0009), patient P/F ratio ≤59 (OR = 8.65, p = .001), presence during ECG (OR = 3.52, p = .002), and presence during intubation OR = 2.79, p = .004).

**Table 4:** Multivariate Generalized Estimating Equation logistic regression model of the probability of transmitting SARS from patient to health care worker.

Parameter	OR	95% CI	p value
HCW's eye/mucous membranes exposed to body fluids	7.34	(2.19, 24.52)	.001
Patient APACHE II score ≥20	17.05	(3.20, 90.75)	.009
HCW present during ECG	3.52	(1.58, 7.86)	.002
HCW present during intubation	2.79	(1.40, 5.58)	.004
Patient PaO_2_ to FiO_2_ ratio ≤59	8.65	(2.31, 32.36)	.001

HCW = health care worker.

## Discussion

This study is the most detailed assessment to date of the risks of SARS acquisition associated with HCW involvement in medical procedures, their infection control practices and the demographic and clinical characteristics of SARS patients. [4]–[6], [14]–[19] Amongst this group of HCWs caring for SARS patients immediately prior to and during intubation, the strongest predictor of SARS transmission from patient to HCW was whether or not the patient under care was a “superspreader”. Twenty-three of the 26 HCWs who became infected with SARS were infected by four of 45 patients. Lack of adherence to infection control procedures was also associated with transmission of SARS-CoV; however, individual patient characteristics beyond superspreader status were not.

The substantial heterogeneity in the number of secondary infections created by each case for many sexually transmitted and vector-borne diseases has led to the general rule that 20% of patients are associated with 80% of new cases. Although heterogeneity in individual patient transmission in diseases spread by direct contact and respiratory droplets is less well recognized, it has been clearly described for measles [20], rubella [21], [22], *Staphylococcus aureus* [23], and tuberculosis [24]–[26], as well as for SARS-CoV in previous publications. [1], [4], [27]–[30] Understanding this heterogeneity is important, because modeling studies demonstrate that the models incorporating variability in transmission differ substantially from standard outbreak models, and, in these models, individually–targeted interventions are much more effective than untargeted interventions. [31], [32] “Superspreading” appears to be a normal feature of disease transmission, and one that must be understood if we are to effectively prevent the spread of respiratory infection. The presence of heterogeneity in transmission also makes the interpretation of observational cohort data about risk factors for transmission difficult: our analysis illustrates the substantial potential impact of confounding in such cohorts. Observational cohort data may have very limited value for assessing transmission of influenza unless patient transmission heterogeneity can be taken into account.

Our findings with respect to HCW activity risk factors are similar to those of other studies assessing HCW risks unadjusted for patient factors, but illustrate the complexity of analyses of cohort studies in these settings. [15]–[19] The factors associated with SARS in other analyses are somewhat different, but essentially all are related to procedures that bring workers into proximity with a patient's airway for prolonged periods of time, or with unprotected faces. In keeping with data from Teleman et al. [16] our highest estimated HCW risk in GEE models was eye or mucous membrane exposure to body fluids (OR = 7.3), while in CART analysis, the primary HCW related risk factor was whether or not eye protection was worn. This should not be interpreted as meaning that conjunctival contact in particular is a primary mode of spread of SARS CoV: when exposure to droplet spray occurs, is it generally not possible to distinguish exposure to eyes versus other mucous membranes. Absence of eye protection results in exposure of facial skin, and transmission could subsequently be from facial skin to hand to other mucous membrane. It is also possible that absence of eye protection is a marker for reduced adherence to other precautionary measures for which adherence is not adequately captured by self-report.

The range of different types of healthcare providers infected emphasizes that healthcare worker safety is not an issue limited to one profession, or to those workers with less education or control over their workplace situation. The finding in our study and those of others that a relatively small amount of education was associated with significant increases in adherence to precautions and reductions in infection also highlights the fact that, at least in some situations, education alone is enough to provide significant safety benefits. [11], [16] Where possible, hospital planners should consider building plans for “just-in-time” training into pandemic and outbreak responses.

CART and GEE logistic regression were complementary techniques for identifying HCW and patient characteristics most associated with transmission of SARS-CoV. Advantages of CART include the ability to identify interactions between variables by identifying specific combinations of variables which place HCWs at higher risk of acquiring SARS, modeling of nonlinear relationships between the dependent and independent variables, ability to handle numerical data that is highly skewed and categorical data with either ordinal or non-ordinal structures and its ease of interpretation. While logistic regression models are not as flexible in handling this variety of data, they yield odds ratios and p values, which are useful for quantifying risk and measuring the statistical significance of relationships between variables. The CART analyses were comparable with logistic regression GEE models with respect to sensitivity, specificity, and positive and negative predictive values.

There are several limitations to this study. Although we used patient charts to enhance recall, and validated our questionnaire [33], [34], HCW recall of various exposures may have been imperfect due to the stress of caring for SARS patients and the time from exposure to interview, may have been biased by HCW outcomes, or may themselves have introduced biases if some were more accurate or complete than others. While our assumption that patients who were known to have infected other HCWs also infected the three HCWs whose source of infection was unclear had the potential to overestimate the superspreader phenomenon, secondary analyses confirmed our findings. While we might speculate that presence in the room during an ECG identified as a risk factor because other variables incompletely adjusted for duration of time in the room when a patient is deteriorating rapidly, we do not have a satisfactory explanation for why this variable is associated with SARS-CoV transmission. Finally, since the greatest risk of transmission of SARS-CoV occurred in the cohort of HCWs caring for the patient with the lowest PaO_2_ to FiO_2_ (P/F) ratio, the superspreader effect was confounded with the P/F ratio effect, and it is not possible to conclude that low patient P/F ratio is associated with SARS-CoV transmission.

Although some authors have assumed that all viral respiratory infections have the same relative modes of transmission, such that identified risk factors and/or interventions that prevent transmission for one can be assumed to be true for others [35], it is not clear that knowledge about risk factors for SARS coronavirus infection can be directly applied to other diseases such as influenza. It is clear, however, that, during the SARS outbreak, HCW exposures to body fluids occurred frequently and adherence to recommended precautions was often incomplete, putting HCWs at significant risk of infection. Thus, research into the incidence of and risk factors for influenza transmission in acute care hospital settings, and into interventions effective in minimizing transmission, is urgently needed.

## Supporting Information

Table S1 Univariate GEE logistic regression models of the probability of transmission of SARS from patient to HCW, for care provided from 24 hours before to four hours after intubation, Toronto, 2003.
(0.23 MB DOC)
