# Supplementary material for: Risk Factors for SARS Transmission from Patients Requiring Intubation: A Multicentre Investigation in Toronto, Canada
Source: PLoS One. 2010 May 19;5(5):e10717. doi: 10.1371/journal.pone.0010717 (PMC2873403; doi:10.1371/journal.pone.0010717)
Supplement: Table S1 — Univariate GEE logistic regression models of the probability of transmission of SARS from patient to HCW, for care provided from 24 hours before to four hours after intubation, Toronto, 2003. (0.23 MB DOC) [file pone.0010717.s001.doc]

| **Paramete** | **# of cohorts in which SARS transmission occurred^a^** | **# of SARS cases/**  **# of HCW shifts with exposure** | **# of SARS cases/**  **# of HCW shifts without exposure** | **OR** | **95% CI** | **p value** |
| --- | --- | --- | --- | --- | --- | --- |
| ***Patient Characteristics at Admission*** |  |  |  |  |  |  |
| Female | 2 | 3/364 | 23/422 | 0.12 | (0.02, 0.64) | 0.01 |
| Age (odds ratio per year of age) | -^b^ | - | - | 1.02 | (0.97, 1.08) | 0.38 |
| Chronic underlying illness^c^ | 3 | 21/315 | 5/471 | 5.77 | (1.36, 24.5) | 0.02 |
| Diabetes | 3 | 21/315 | 5/504 | 7.52 | (1.78, 31.8) | 0.006 |
| Immunosuppression | 1 | 6/63 | 20/723 | 2.38 | (0.32, 17.7) | 0.40 |
| Patient incontinent | 4 | 10/276 | 16/510 | 1.16 | (0.22, 6.01) | 0.86 |
| Patient PaO_2_ to FiO_2_  ratio ≤ 59 | 3 | 21/228 | 5/558 | 10.65 | (2.56, 44.4) | 0.001 |
| Patient recognized as a SARS case | 6 | 20/683 | 6/103 | 0.69 | (0.09, 5.61) | 0.73 |
| Patient transmitted SARS to prior to at risk period^d^ | 4 | 15/327 | 11/459 | 1.69 | (0.26, 10.8) | 0.58 |
| Patient APACHE II score ≥ 20 | 5 | 24/283 | 2/503 | 18.57 | (3.55, 97.3) | 0.0005 |
| FiO_2_ on day 2 of hospital admission | -^b^ | - | - | 1.41 | (0.10, 19.2) | 0.80 |
| ***Patient/ characteristics at/near intubation****^e^* |  |  |  |  |  |  |
| Patient had diarrhea | 1 | 2/332 | 24/454 | 0.09 | (0.01, 0.72) | 0.02 |
| Patient agitated, combative, or confused | 1 | 8/147 | 18/639 | 2.45 | (0.28, 21.1) | 0.42 |
| Patient had copious respiratory secretions | 1 | 8/256 | 18/530 | 1.04 | (0.12, 9.03) | 0.97 |
| Patient vomited | 1 | 2/122 | 24/664 | 0.30 | (0.04, 2.36) | 0.25 |
| ***Intubation characteristics***  Intubation occurred 5–12 days post symptom onset | 6 | 20/519 | 6/267 | 2.42 | (0.28, 21.1) | 0.42 |
| Intubation performed during night (12am–7am) | 1 | 1/137 | 25/649 | 0.24 | (0.03, 1.95) | 0.18 |
| Difficult intubation | 3 | 16/254 | 10/532 | 2.87 | (0.48, 17.1) | 0.25 |
| Emergency intubation | 1 | 1/15 | 25/771 | 2.53 | (0.97, 6.57) | 0.06 |
| Intubation performed by experienced physician | 6 | 18/695 | 8/91 | 0.32 | (0.04, 2.71) | 0.29 |
| Intubation performed in negative pressure room | 6 | 20/687 | 6/99 | 0.68 | (0.08, 5.44) | 0.71 |
| Patient required high frequency ventilation | 0 | 0/276 | 26/786 | n/a^f^ |  |  |
| ***HCW Characteristics*** |  |  |  |  |  |  |
| Profession |  |  |  |  |  |  |
| Respiratory therapist | 2 | 4/119 |  | 1.17 | (0.06, 21.3) | 0.92 |
| Registered nurse | 6 | 11/330 |  | 1.49 | (0.23, 9.85) | 0.68 |
| Medical doctor (staff or resident) | 2 | 6/114 |  | 1.97 | (0.22, 17.9) | 0.55 |
| Radiologist | 1 | 1/88 |  | 1.19 | (0.12, 12.3) | 0.88 |
| Other | 2 | 4/109 |  | 1.00 | ref |  |
| Underlying chronic illness | 1 | 1/43 | 25/724 | 0.94 | (0.24, 3.59) | 0.92 |
| Number of hours worked in previous 7 days | -^b^ | - | - | 1.01 | (0.99, 1.04) | 0.40 |
| Infection control training |  |  |  |  |  |  |
| Any infection control training | 4 | 6/224 | 20/562 | 0.68 | (0.16, 2.94) | 0.61 |
| Passive infection control training | 1 | 2/178 |  | 0.36 | (0.12, 1.13) | 0.08 |
| Active infection control training | 4 | 8/376 |  | 0.28 | (0.08, 1.02) | 0.053 |
| No infection control training | 5 | 16/206 |  | 1.00 | ref |  |
| Always wore recommended PPE^g^ | 5 | 12/602 | 14/184 | 0.70 | (0.19, 2.58) | 0.59 |
| Always wore eye protection while in the room | 5 | 13/603 | 13/183 | 0.90 | (0.16, 5.02) | 0.91 |
| Always wore gloves while in the room | 7 | 23/737 | 3/28 | 1.49 | (0.72, 3.11) | 0.28 |
| Always wore gown while in the room | 7 | 20/719 | 6/67 | 0.60 | (0.16, 2.24) | 0.45 |
| Removal of PPE  No hand hygiene described | 4 | 11/240 |  | 0.87 | (0.16, 4.65) | 0.87 |
| Hand hygiene performed once | 4 | 8/400 |  | 0.67 | (0.11, 3.99) | 0.66 |
| Adequate PPE removal^h^ | 3 | 4/77 |  | 1.18 | (0.20, 6.83) | 0.85 |
| Number of times HCW entered patient’s room |  |  |  |  |  |  |
| 1 - 2 times | 5 | 12/390 |  | 0.67 | (0.28, 1.63) | 0.38 |
| 3 - 5 times | 4 | 6/172 |  | 0.69 | (0.39, 1.23) | 0.21 |
| 6 – 10 times | 3 | 3/103 |  | 0.41 | (0.14, 1.20) | 0.10 |
| > 10 times | 5 | 5/121 |  | 1.00 | ref |  |
| Duration of face to face contact with patient |  |  |  |  |  |  |
| < 1 minute | 2 | 2/96 |  | 0.83 | (0.11, 6.27) | 0.86 |
| 1-10 minutes | 3 | 7/245 |  | 0.98 | (0.26, 3.71) | 0.98 |
| 11-30 minutes | 4 | 6/161 |  | 1.33 | (0.20, 8.88) | 0.77 |
| 31-60 minutes | 2 | 6/85 |  | 2.73 | (0.33, 22.5) | 0.35 |
| 1-4 hours | 3 | 4/77 |  | 2.37 | (0.41, 13.6) | 0.33 |
| > 4 hours | 1 | 1/51 |  | 1.00 | ref |  |
| ***Documented exposure to body fluids*** |  |  |  |  |  |  |
| Eye/mucous membranes splash | 2 | 4/10 | 22/776 | 6.90 | (1.06, 44.9) | 0.04 |
| Bare skin exposure to blood or body fluid | 1 | 2/18 | 24/768 | 1.75 | (0.15, 20.7) | 0.65 |
| Skin touched patient’s mucous membranes | 4 | 11/259 | 15/527 | 1.55 | (0.84, 2.86) | 0.16 |
| ***Potential HCW Exposure to Respiratory Secretions^i^*** |  |  |  |  |  |  |
| Non-invasive ventilation | 3 | 10/120 | 16/666 | 1.37 | (0.14, 13.1) | 0.78 |
| Bronchoscopy | 0 | 0/11 | 26/775 |  |  |  |
| Cardiac compressions | 1 | 1/9 | 25/777 | 6.39 | (3.27, 12.5) | <0.0001 |
| Chest physiotherapy | 1 | 1/56 | 25/730 | 1.03 | (0.35, 3.04) | 0.95 |
| Defibrillation | 1 | 1/4 | 25/782 | 15.73 | (5.27, 46.9) | <0.0001 |
| High flow oxygen | 2 | 2/118 | 24/668 | 0.67 | (0.43, 1.04) | 0.07 |
| Mechanical ventilation | 3 | 9/276 | 17/510 | 1.06 | (0.49, 2.30) | 0.87 |
| Nebulizer treatment | 0 | 0/9 | 26/777 |  |  |  |
| Collection of sputum sample | 3 | 4/46 | 22/740 | 3.25 | (2.11, 5.01) | <0.0001 |
| Insertion of NG tube | 2 | 2/55 | 24/731 | 1.55 | (0.72, 3.32) | 0.26 |
| ***Procedures with potential exposure to urine or stool^i^*** |  |  |  |  |  |  |
| Emptying urine bag or taking urine sample | 3 | 4/159 | 22/627 | 0.97 | (0.53, 1.78) | 0.92 |
| Emptying bed pan | 1 | 1/53 | 25/733 | 0.90 | (0.66, 1.23) | 0.52 |
| Collection of stool sample | 2 | 2/21 | 24/765 | 2.64 | (1.40, 4.97) | 0.003 |
| ***Activities related to intubation*** |  |  |  |  |  |  |
| HCW intubated the patient | 2 | 3/48 | 23/738 | 2.98 | (1.19, 7.48) | 0.02 |
| Present during suctioning before intubation | 4 | 7/129 | 19/657 | 1.78 | (0.97, 3.29) | 0.06 |
| Present during suctioning after intubation | 4 | 10/196 | 16/590 | 1.63 | (0.97, 2.73) | 0.07 |
| Present during manual ventilation after intubation | 4 | 6/133 | 20/653 | 1.83 | (1.09, 3.07) | 0.02 |
| Present during manual ventilation before intubation | 4 | 10/137 | 16/649 | 2.68 | (1.34, 5.35) | 0.005 |
| Present in room during intubation | 4 | 12/163 | 14/623 | 2.92 | (1.70, 5.03) | 0.0001 |
| ***Other patient care activities^i^*** |  |  |  |  |  |  |
| Insertion of central venous line | 2 | 3/63 | 23/723 | 1.50 | (0.83, 2.69) | 0.18 |
| Chest tube insertion | 0 | 0/12 | 26/774 |  |  |  |
| Insertion of peripheral intravenous access line | 4 | 7/160 | 19/626 | 1.57 | (0.42, 5.90) | 0.50 |
| Insertion urinary catheter | 2 | 3/42 | 23/744 | 2.51 | (0.95, 6.66) | 0.06 |
| Venipuncture/arterial blood gas | 3 | 7/194 | 19/592 | 1.45 | (0.58, 3.63) | 0.43 |
| ECG | 4 | 11/124 | 15/662 | 4.26 | (2.05, 8.87) | 0.0001 |
| Bathing a patient | 3 | 4/158 | 22/628 | 0.79 | (0.30, 2.10) | 0.64 |
| Feeding a patient | 1 | 1/96 | 25/690 | 0.43 | (0.07, 2.53) | 0.35 |
| Administering oral medication | 2 | 3/126 | 23/660 | 0.65 | (0.20, 2.06) | 0.46 |
| Taking oral temperature | 2 | 2/81 | 24/705 | 0.84 | (0.19, 3.68) | 0.82 |
| Patient transportation | 5 | 7/106 | 19/680 | 3.32 | (1.17, 9.46) | 0.02 |
| ***Housekeeping Activities^i^*** |  |  |  |  |  |  |
| Cleaning bathroom | 1 | 1/53 | 25/733 | 0.87 | (0.13, 5.89) | 0.88 |
| Cleaning equipment | 6 | 7/198 | 19/588 | 1.08 | (0.32, 3.65) | 0.90 |
| Cleaning room | 1 | 2/104 | 24/682 | 0.39 | (0.01, 18.3) | 0.63 |
| Changing bedding | 4 | 7/209 | 19/577 | 1.05 | (0.43, 2.54) | 0.91 |

^a^Overall, transmission occurred in 7 of 45 cohorts

^b^Not applicable, variable is continuous

^c^Chronic underlying illness is defined as having one or more of diabetes, chronic renal failure, chronic liver disease, chronic obstructive pulmonary syndrome, coronary artery disease, congestive heart failure, active cancer, HIV/AIDS, transplantation.

^d^Transmission occurred from patient to household or hospital contact prior to study period (starting 24 hours prior to intubation)

^e^Characteristics present in the 24 hours prior to intubation

^f^Odds ratio cannot be calculated

^g^Recommended personal protective equipment (PPE) during the outbreak was gown, gloves, face shield or goggles, and N95 (or higher) respirator

^h^Adequate PPE removal was defined as removal in which contaminated hands were never in contact with face (see reference 11)

^i^For this analysis, HCWs were included if they were present in the room during the procedure/activity (ie. performed, assisted, or observed the
